# Association Between Eating Speed and Metabolic Syndrome: A Systematic Review and Meta-Analysis

**DOI:** 10.3389/fnut.2021.700936

**Published:** 2021-10-20

**Authors:** Shu-qian Yuan, Ying-ming Liu, Wei Liang, Fei-fei Li, Yuan Zeng, Yin-yue Liu, Shu-zhen Huang, Quan-yuan He, Binh Quach, Jiao Jiao, Julien S. Baker, Yi-de Yang

**Affiliations:** ^1^Key Laboratory of Molecular Epidemiology of Hunan Province, School of Medicine, Hunan Normal University, Changsha, China; ^2^Centre for Health and Exercise Science Research, Hong Kong Baptist University, Hong Kong, China; ^3^Department of Sport, Physical Education and Health, Hong Kong Baptist University, Hong Kong, China; ^4^Dr. Stephen Hui Research Centre for Physical Recreation and Wellness, Hong Kong Baptist University, Hong Kong, China

**Keywords:** metabolic syndrome, central obesity, eating speed, meta-analysis, systematic review, elevated blood pressure

## Abstract

**Objective:** This review aimed to systematically summarize and meta-analyze the association between eating speed and metabolic syndrome (MetS).

**Methods:** Following the Preferred Reporting Items for Systematic Reviews, and Meta Analyses (PRISMA) guidelines, four electronic databases (PubMed, Web of Science, MEDLINE, and EMBASE) were searched until March 2021 to identify eligible articles based on a series of inclusion and exclusion criteria. Heterogeneity was examined using *I*^2^ statistics. Using random-effects models, the pooled odds ratios (ORs), and 95% CIs were calculated to evaluate the association between eating speed with MetS and its components, including central obesity, blood pressure (BP), high-density lipoprotein cholesterol (HDL), triglyceride (TG), and fasting plasma glucose (FPG).

**Results:** Of the 8,500 original hits generated by the systematic search, 29 eligible studies with moderate-to-high quality were included, involving 465,155 subjects. The meta-analysis revealed that eating faster was significantly associated with higher risks of MetS (OR = 1.54, 95% CI: 1.27–1.86), central obesity (OR = 1.54, 95% CI: 1.37–1.73), elevated BP (OR = 1.26, 95% CI: 1.13–1.40), low HDL (OR = 1.23, 95% CI: 1.15–1.31), elevated TG (OR = 1.29, 95% CI: 1.18–1.42), and elevated FPG (OR = 1.16, 95% CI: 1.06–1.27) compared to eating slowly.

**Conclusions:** The results of the review indicated that eating speed was significantly associated with MetS and its components. Interventions related to decreasing eating speed may be beneficial for the management of MetS.

**Systematic Review Registration:** https://www.crd.york.ac.uk/prospero/display_record.php?ID=CRD42021242213, identifier: CRD42021242213.

## Introduction

Metabolic syndrome (MetS) is a cluster of metabolic abnormalities characterized by central obesity, elevated blood pressure (BP), low high-density lipoprotein cholesterol (HDL), elevated triglyceride (TG), and elevated fasting plasma glucose (FPG) (1, 2). MetS is not only a risk factor for cardiovascular disease and type 2 diabetes, but is also associated with colorectal cancer, pancreas, and primary liver cancer (3–7). MetS is a major public health threat in modern societies (8).

The prevalence of MetS is increasing globally in both developed and developing countries (9). A National Health and Nutrition Survey (NHANES) in the US showed that the estimated population of obesity and diabetes increased by 20.4 million and 9 million, respectively, from 2003–2004 to 2013–2014 (10). The prevalence of MetS reported was 34.7%, even higher in adults aged 60 years above to 48.6% in the US (1). Several studies summarized the prevalence of MetS in Asia [such as, Iran (2009–2017: 31%), Thailand (2013: 18.2%), Central India (2017: 50.2%), Malaysia (2018: 24.2%), China (2017: 25.59%), and Korea (2015: 31.7%)] (11–16). This lower prevalence of MetS in Asian populations, compared with Americans, maybe due to the higher prevalence of obesity in American and Western populations (17). Nevertheless, the prevalence of MetS in Asia is still on the increase (18).

Dietary behavior, such as eating speed, is a modifiable factor to control the risk of MetS and its components. Previous studies have indicated that unhealthy eating habits, such as overeating, skipping breakfast, and preference for fried foods, were all risk factors for the occurrence of MetS (19–21). Recommendations on adequate intake of micro- or macronutrients and energy requirements have been established using different guidelines, but the appropriate eating speed for the prevention of MetS is still uncertain. A study conducted in Japan demonstrated that eating fast would increase the risk of developing MetS (22). It was shown that fast-eating speed was associated with a high risk of developing MetS, elevated BP, and central obesity in both genders among Chinese populations (23). Nanri et al. found that eating quickly was positively correlated with an increased likelihood of central obesity, but was not correlated with high BP, high FPG, high TG, and low HDL-C (24). In summary, the association between eating speed with the risk of MetS and its components is inconsistent. Given the epidemic and detrimental impact of MetS, improving our understanding of the precise estimation of the association between eating speed and MetS would be beneficial from a public health care perspective.

Therefore, the purpose of this study was to conduct a systematic review and meta-analysis to assess the association between eating speed and MetS. A further aim was to estimate the pooled odds ratios (ORs), to investigate further any relationships between eating speed and MetS and its components.

## Methods

### Data Sources and Search Strategy

Relevant literature searching was conducted following the Preferred Reporting Items for Systematic Reviews, and Meta Analyses (PRISMA) statement (25, 26). The current study was registered on PROSPERO International Prospective Register of Systematic Reviews (CRD42021242213, April 11, 2021, https://www.crd.york.ac.uk/PROSPERO/).

We comprehensively searched PubMed, Web of Science, Medline, and Embase databases from their establishment to March 2021. The search terms were based on subheadings related to MetS (i.e., “metabolic syndrome” or “central obesity” or “abdominal obesity” or “high density lipoprotein” or “blood pressure” or “SBP” or “DBP” or “hypertension” or “triglyceride” or “HDL” or “FPG”) and eating speed [i.e., (“meal” or “eating” or “eat” or “eater”) and (“rapid” or “fast” or “quick” or “quickly” or “speed” or “rate” or “slow” or “time” or “duration”)]. The detailed search strategies are available as (see Supplementary File 1). We also manually recorded the reference lists of the identified papers to obtain possible relevant studies.

### Eligibility and Study Selection

Two authors (SQY and YML) selected and evaluated articles independently; disagreements were solved by consultation with another senior author (YDY). For the literature search, we used the Population, Intervention, Control, Outcome, and Study approach (PICOS) to define the study eligibility criteria: (1) Population (P): general population; (2) Interventions or exposure (I): observational studies were eligible if they provided comparisons of groups from different eating speed. In addition, randomized controlled trials (RCTs) were eligible if the intervention of eating speed clearly appears in the study and the randomization method is clarified, and if available the baseline data of RCTs were extracted for analysis; (3) Control or comparators (C): studies that compared participants with different eating speed; (4) Outcomes (O): MetS or its components (such as, central obesity, high BP, high FPG, high TG, and low HDL), (5) Study design (S): cross-sectional studies, cohort studies, case-control studies, or RCTs. The exclusion criteria were as follows: review articles or abstracts, no data of interest, animal experiments, papers where full information cannot be accessed, or the methodologies were recognized as weak.

### Data Extraction

From the included articles, the following information was extracted: (1) title and year of publication; (2) name of the first author; (3) area; (4) study design; (5) statistical model; (6) gender ratio of the population; (7) sample size; (8) population (adults or children); (9) mean or median of the age; (10) definition of MetS; (11) risk estimates (adjusted OR and 95% CI); (12) outcome variables; (13) main result; and (14) and list of adjusted covariates. The primary outcome measure was MetS in the present study. Secondary outcomes of interest included different components of MetS, such as central obesity, elevated BP, FPG, TG, and low HDL.

### Quality Assessment

The Agency for Healthcare Research and Quality (AHRQ) scale was used to assess cross-sectional studies and scores of “0–3,” “4–7,” and “8–11” were defined as low, moderate, high quality (27). The Newcastle-Ottawa Scale (NOS) was used to assess cohort and case-control studies, and scores of “0–3,” “4–6,” and “7–9” were defined as low, moderate, and high quality (28). The Cochrane assessment scale was used to assess RCTs (29). The quality assessment forms are available from: https://www.ncbi.nlm.nih.gov/books/NBK35156/.

### Statistical Analysis

In the included studies, eating speed was measured by questionnaire, including professional questionnaires, namely, food frequency questionnaire (FFQ), brief diet history questionnaire (BDHQ), or self-designed questionnaire. The classification of eating speed was self-designed without unified criteria, and participants chose an option in the classification that they thought was consistent with their own situation compared to other participants. The number of options for the eating speed question is different. There are four classification methods based on the number of options of the eating speed question in the included studies: (1) two options: slow and fast eating; (2) three options: slow, medium, and fast eating; (3) four options: slow, medium, fast, and very fast eating; (4) five options: very slow, slow, medium, fast, and very fast eating. The validity of the questionnaire was confirmed in 16 studies and was not mentioned in the rest of the studies. The associations between eating speed and risk of MetS, central obesity, elevated TG, low HDL, elevated BP, and elevated FPG were estimated by the pooled OR with 95% CI. The heterogeneity of studies was calculated using the *I*^2^ statistic, and the random-effects model was used in this calculation, *I*^2^ ≥ 50% or *P* < 0.10 was defined as significant heterogeneity (30, 31). Additionally, sensitivity analyses, Begg's funnel plot, and Egger's test were used to estimating possible contributing factors of heterogeneity and potential publication bias (32–34). A statistically significant difference was defined as *P* < 0.05. Studies with no analyzable data were discussed only in the systematic review. We conducted subgroup analysis according to age, gender, location, eating speed, and study design. Subsequently, meta-regression analysis was also conducted to estimate the source of heterogeneity. Most studies used the slow-eating group as the reference group and provided the adjusted OR and 95% CI for the non-slow-eating groups. If the slow-eating speed group was not the reference group in the included study, we performed transformation to calculate the appropriate OR for meta-analysis so as to guarantee comparisons and calculation of pooled OR. We used R software 3.3 (R Development Core Team, Vienna, Austria) with Meta-Analysis Package for R (meta) to conduct subgroup analysis, sensitivity analyses, Begg's funnel plot, and Egger's test. Stata 11.0 (College Station, TX, USA) was used for meta-regression analysis. In addition, Review Manager 5.4 (The Nordic Cochrane Centre, The Cochrane Collaboration, Copenhagen, Denmark) was used for the meta-analyses and heterogeneity tests.

## Results

### Descriptions of Literature Search

**Figure 1:**
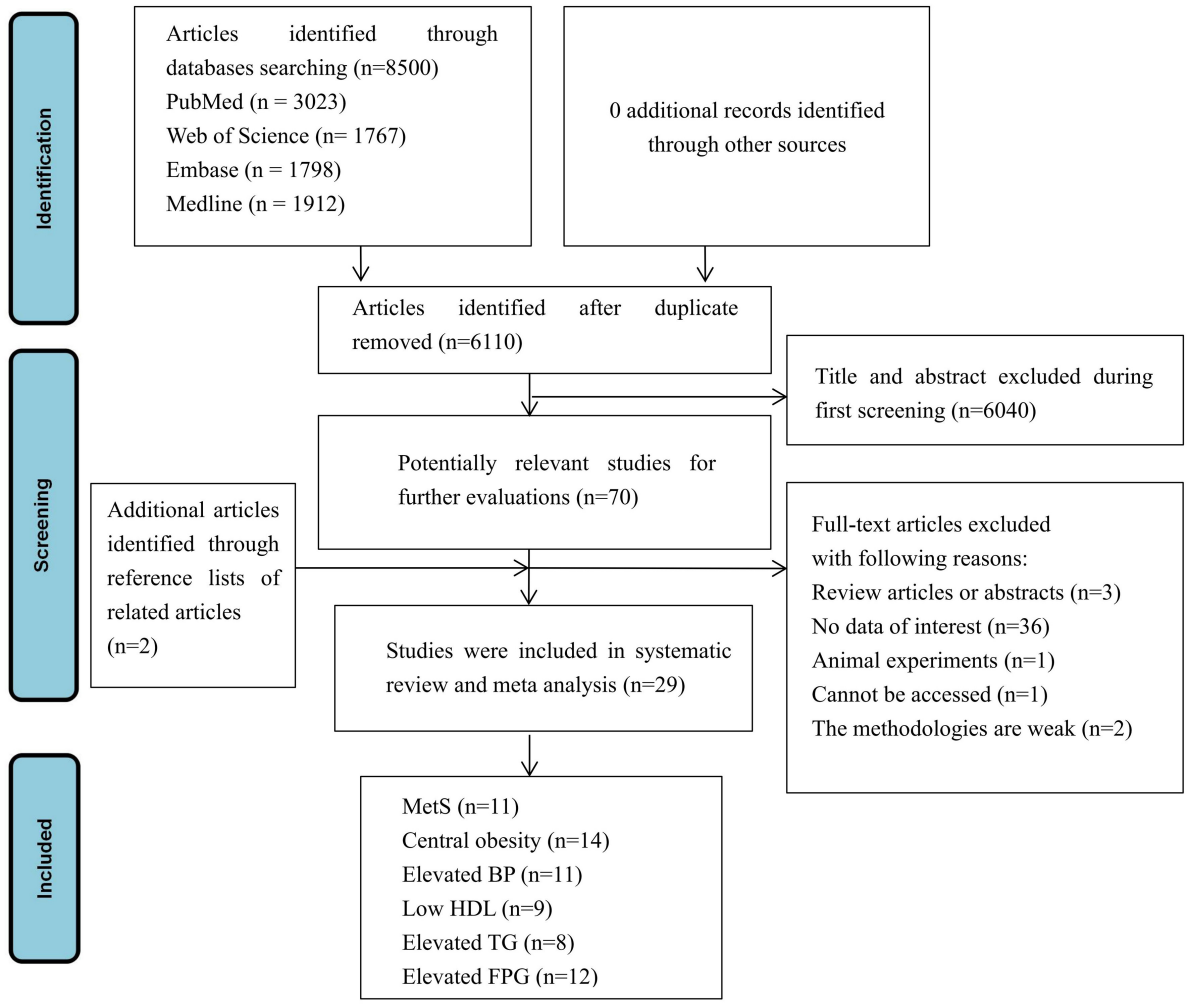
Flow chart of the selection process of the eligible studies. MetS, metabolic syndrome; BP, blood pressure; FPG, fasting plasma glucose; TG, triglycerides; HDL, high-density lipoprotein.

A total of 8,500 articles were initially identified from the electronic database. Of those, 2,390 duplicates were subsequently excluded either manually or automatically; then, the remaining 6,110 articles were screened for relevance based on title and abstract, resulting in the removal of 6,040 articles. According to the standard screening procedure, 70 articles were identified for in depth reading with the full-texts. Two articles were included through reference lists of related articles. After reading the full texts, a total of 43 articles were excluded, i.e., 40 articles for not meeting the eligibility criteria and 3 articles for full information that cannot be accessed, or the methodologies were recognized as weak. Finally, 29 articles were selected for the systematic review and meta-analysis. Eleven studies (22–24, 35–42) examining the association between eating speed and the risk of MetS were included into the systematic review, of which 7 (23, 35–40) provided with enough data and were included into the quantitative synthesis. Fourteen studies (23, 24, 37, 39, 43–52) examining the association between eating speed and the risk of central obesity were included into the systematic review, of which 11 (23, 24, 37, 39, 44–50) provided with enough data and were included into the quantitative synthesis of the meta-analysis. Eleven studies (23, 24, 37, 39, 42, 43, 47, 50, 53–55) examining the association between eating speed and the risk of elevated BP were included into the systematic review, of which 6 (23, 24, 37, 39, 50, 55) were included into the quantitative synthesis of the meta-analysis. Nine studies (23, 24, 37, 39, 43, 50, 53, 55, 56) examining the association between eating speed and the risk of low HDL were included into the systematic review, of which 6 (23, 24, 37, 39, 50, 55) were included into the quantitative synthesis of the meta-analysis. Eight studies (23, 24, 37, 39, 43, 50, 53, 55) examining the association between eating speed and the risk of elevated TG were included into the systematic review, of which 6 (23, 24, 37, 39, 50, 55) were included into the quantitative synthesis of the meta-analysis. Twelve studies (23, 24, 37, 39, 42, 50, 53, 55, 57–60) examining the relationship between eating speed and elevated FPG, of which 9 for meta-analysis (23, 24, 37, 39, 50, 55, 58–60) were included into the quantitative synthesis of the meta-analysis. The selection process for eligible articles is outlined in Figure 1.

### Study Characteristics

The overall study characteristics are shown in Table 1, 29 relevant studies were published from 2007 to 2021, the sample size ranged from 106 to 197,825. The study population included children and adults (aged from 6 to 99 years). Among the 29 relevant studies, 20 were cross-sectional studies, 7 were cohort studies, 1 RCT, and 1 case-control study. In these studies, 16 were conducted in Japan, four in Korea, two in China, two in Spain, one in Singapore, one in Malaysia, one in England, one in America, and one in Iran. Adjusted covariates included age, gender, ethnicity, education level, total physical activity, smoking, alcohol consumption status, and body mass index (BMI), and so on. The detailed information of covariates extracted from the included studies is shown in Supplementary Table 1. The detailed information extracted from the included studies is shown in Supplementary Table 1. The quality of articles is provided in Supplementary Tables 2–4. The results showed that 29 articles included in this study were of medium or high quality, in which the 20 cross-sectional studies with AHRQ scores (Supplementary Table 2) ranged from 4 to 8, and 7 cohort studies and 1 case-control study with the The Newcastle-Ottawa Scale (NOS) scores (Supplementary Table 3) ranged from 6 to 9. The results of Begg's test and Egger's test of the included studies are shown in Supplementary Figure 1, Supplementary Table 5. No publication bias was found excluding central obesity (Supplementary Figure 1, Supplementary Table 5). The results of sensitivity analysis are shown in Supplementary Figure 2.

**Table 1:** The overall characteristics of the studies reporting on the association between eating speed and metabolic syndrome and its components.

**Characteristic**	**Number of studies**	**Percentages (%)**
**Total samples (*****N*** **= 29)**		
**Location**		
Japan	16	55.17
Korea	4	13.79
China	2	6.89
Singapore	1	3.45
Malaysia	1	3.45
Iranian	1	3.45
Spain	2	6.89
England	1	3.45
America	1	3.45
**Study design**		
Cohort study	7	24.14
Cross-sectional study	20	68.97
Others	2	6.89
**Age**		
Children (6–18 yr)	5	17.24
Adults (18–99 yr)	24	82.76
**Gender**		
All female	1	3.45
All male	4	13.79
Male and Female	24	82.76
**Classification of eating speed**		
2 groups	12	41.38
3 groups	12	41.38
4 groups	4	13.79
5 groups	1	3.45
**Risk of bias assessment**		
High quality	9	31.03
Moderate quality	20	68.97
Low quality	0	0.0
**Reference group**		
Slow eating speed	18	62.07
Moderate eating speed	4	13.79
Others	7	24.14

### MetS

Eleven studies examining the relationship between eating speed and MetS were all included in the systematic review (22–24, 35–42). These 11 studies were all conducted in adults. Although two cross-sectional studies (38, 39) did not find a statistically significant association between eating speed and risk of MetS. Eight articles (23, 24, 35, 36, 39–42) showed a significantly higher risk in those who had faster-eating habits compared with those who had a slow-eating habit, among which two (24, 35) articles indicated the significantly increased risk of MetS in the fast-eating group, compared with the medium-eating group. Compared with the slow-eating group, five articles (23, 36, 40–42) showed a higher risk of MetS in the faster-eating group among adults. In addition, one article (39) reported a cohort study that enrolled 8,941 participants from 2008 to 2011 showing a significantly higher incidence of MetS in the fast-eating group compared with the non-fast-eating group. One cohort study (22) indicated that slow-eating speed reduced the risk of developing MetS significantly compared with a faster-eating speed group.

**Figure 2:**
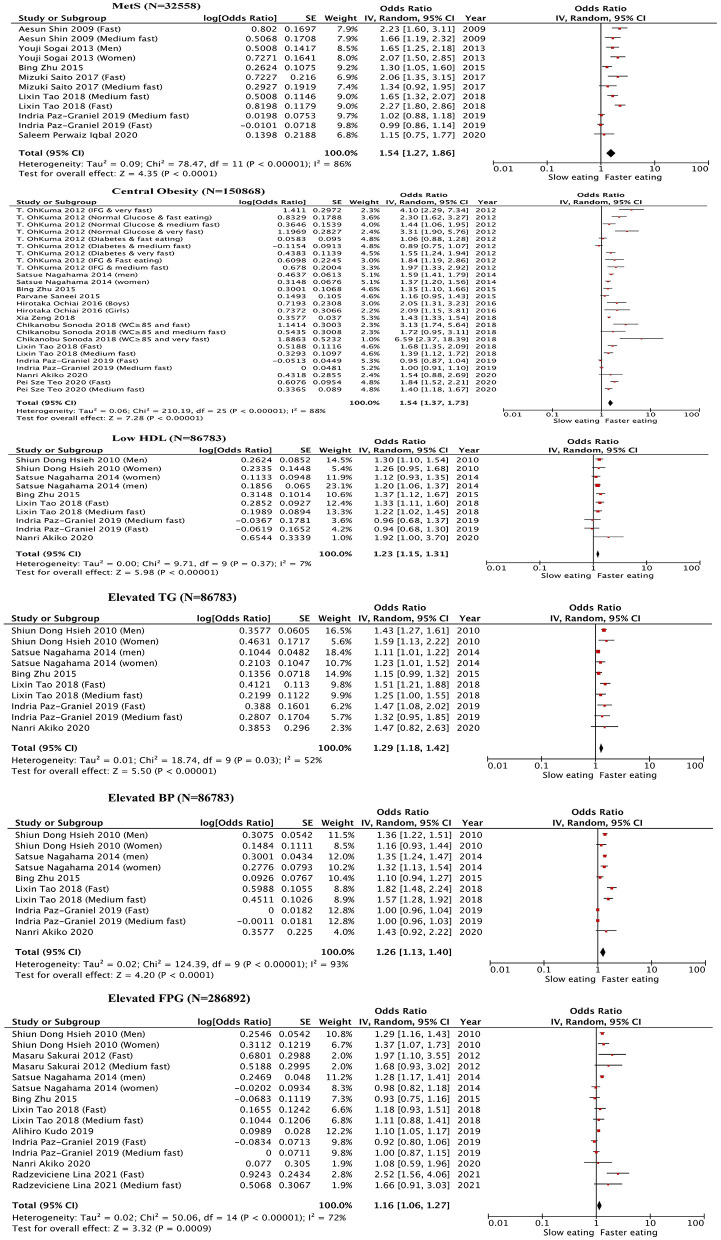
Forest plot of the odds ratios for metabolic syndrome and its components in association with eating rate (The slow eating group were used as the reference group. MetS, metabolic syndrome; BP, blood pressure; FPG, fasting plasma glucose; TG, triglycerides; HDL, high-density lipoprotein).

As shown in Figure 2, seven studies (23, 35–40) were included in the meta-analysis. Compared to the slow-eating speed group, subjects in the faster-eating eating had a significantly higher risk of MetS (OR = 1.54; 95% CI: 1.27–1.86). In addition, in the subgroup analysis, the pooled OR of the risk of MetS for the fast-eating speed group was 1.63 (95% CI: 1.26–2.12) (23, 37, 39, 40, 55), OR for the risk for medium-eating speed group was 1.36 (95% CI: 1.00–1.86; Table 2) (23, 24, 37, 40, 50).

**Table 2:** Subgroup analysis based on age, gender, location, eating speed, and study design.

**Outcomes**	**Subgroup**		**Included studies**	**OR**	**95%CI**	***I* ^**2**^**	***P***	**Overall**	
								***I* ^**2**^**	***P***
MetS	Location	Asian	[23, 35, 36, 38–40]	1.70	1.47–1.98	0.59	<0.01	0.86	<0.01
(*n* = 32,558)		Non–Asian	[37]	1.00	0.91–1.11	0.00	0.77		
	Speed	Fast	[23, 35–40]	1.63	1.26–2.12	0.87	<0.01		
		Medium	[23, 35–37]	1.36	1.00–1.86	0.84	<0.01		
	Study design	Cross–sectional study	[23, 35–38, 40]	1.56	1.26–1.94	0.87	<0.01		
		Cohort study	[39]	1.30	1.05–1.60	0.86	<0.01		
Central obesity	Age	Children	[44, 48]	1.65	1.25–2.19	0.48	0.15	0.88	<0.01
(*n* = 150,868)		Adult	[23, 24, 37, 39, 45–47, 49, 50]	153	1.34–1.75	0.89	<0.01		
	Gender	Male and Female	[23, 24, 37, 39, 44, 45, 47–50]	1.48	1.32–1.66	0.88	<0.01		
		All male	[46]	2.97	1.52–5.80	0.63	0.07		
	Location	Asian	[23, 24, 39, 44–50]	1.59	1.43–1.76	0.78	<0.01		
		Non–Asian	[37]	0.97	0.91–1.04	0.00	0.44		
	Speed	Very–fast	[46, 47]	3.14	1.62–6.09	0.84	<0.01		
		Fast	[23, 37, 39, 44–47]	1.65	1.31–2.07	0.89	<0.01		
		Medium	[23, 24, 37, 45–50]	1.32	1.15–1.52	0.88	<0.01		
	Study design	Cross-sectional study	[23, 37, 39, 41–47]	1.62	1.43–1.83	0.88	<0.01		
		Cohort study	[24, 39]	1.37	1.13–1.66	0.00	0.67		
Elevated BP	Location	Asian	[23, 24, 39, 50, 55]	1.36	1.23–1.49	0.64	<0.01	0.93	<0.01
(*n* = 86,783)		Non-Asian	[37]	1.00	0.82–1.22	0.00	1.00		
	Speed	Fast	[23, 37, 39, 55]	1.25	1.02–1.52	0.93	<0.01		
		Medium	[23, 24, 37, 50]	1.30	1.06–1.59	0.94	<0.01		
	Study design	Cross-sectional study	[23, 24, 37, 50, 55]	1.27	1.13–1.43	0.94	<0.01		
		Cohort study	[39]	1.15	0.95–1.40	0.18	0.27		
Elevated FPG	Gender	Male and Female	[23, 24, 37, 39, 50, 55, 59, 60]	1.14	1.04-1.25	0.70	<0.0	0.72	<0.01
(*n* = 286,892)		All male	[58]	1.82	1.20–2.76	0.00	0.71		
	Location	Asian	[23, 24, 39, 50, 55, 59, 60]	1.17	1.08–1.28	0.61	<0.01		
		Non–Asian	[37, 58]	1.25	0.92–1.68	0.84	<0.01		
	Speed	Fast	[23, 37, 39, 55, 58–60]	1.20	1.06–1.36	0.80	<0.01		
		Medium	[23, 24, 37, 50, 58, 60]	1.14	0.99–1.31	0.60	0.02		
	Study design	Cross–sectional study	[23, 37, 55, 58, 60]	1.16	1.04–1.30	0.73	<0.01		
		Cohort study	[24, 39, 59]	1.08	1.00–1.16	0.07	0.34		
		Others	[60]	2.13	1.43–3.18	0.12	0.29		
Low HDL	Speed	Fast	[23, 37, 39, 55]	1.29	1.17–1.42	0.00	0.5	0.07	0.37
(*n* = 86,783)		Medium	[23, 24, 37, 50]	1.17	1.07–1.29	0.11	0.34		
	Study design	Cross–sectional study	[23, 37, 50, 55]	1.21	1.13–1.29	0.00	0.48		
		Cohort study	[24, 39]	1.41	1.16–1.71	0.00	0.33		
Elevated TG	Speed	Fast	[23, 37, 39, 55]	1.38	1.22–1.56	0.51	0.0	0.52	0.03
(*n* = 86,783)		Medium	[23, 24, 37, 50]	1.34	1.12–1.61	0.00	0.59		
	Study design	Cross–sectional study	[23, 37, 50, 55]	1.17	1.02–1.34	0.00	0.42		
		Cohort study	[24, 39]	1.32	1.19–1.47	0.58	0.02		

*MetS, metabolic syndrome; BP, blood pressure; FPG, fasting plasma glucose; HDL, high-density lipoprotein; TG, triglyceride*.

### Central Obesity

Fourteen studies examining the relationship between eating speed and central obesity were included in our systematic review (23, 24, 37, 39, 43–52). In these studies, 11 studies involved adults, and 3 studies involved children (44, 48, 51). Two studies (39, 44) used slow-eating speed as the control group and divided eating speed into two groups, one (44) (*N* = 2,136) was conducted in Japan and was a cross-sectional study in children (age: 12–13 years), waist-to-height ratio (WHtR) was used as an outcome variable in this study. The study found that eating quickly led to an increased risk of central obesity (defined as WHtR ≥ 0.5) in boys and girls. The second study (39) (*N* = 8,941) was a cohort study conducted among adults in Japan and China, this study used waist circumference (WC) as the outcome variable and demonstrated that the risk in the fast-eating group significantly increased compared with the reference group.

Eating speed was divided into three groups in seven studies (23, 24, 37, 45, 48–50). Three (23, 37, 45) regarded the slow-eating group as a reference group, though one study (37) conducted in Spain (*N* = 792) did not find a significantly increased risk for central obesity, two studies (23, 45) conducted in Singapore and China indicated that the risk increased significantly among adults who ate fast. Four studies (24, 48–50) regarded the moderate-eating group as the reference group. One study (24) conducted in Japan (*N* = 1,018) indicated that fast-eating speed was associated with an increased likelihood of developing central obesity. Similar results were found in a study (39) conducted among Japanese adults. One study (49) conducted in Iran (*N* = 7,958) did not demonstrate significant associations between eating speed and central obesity. One study (48) (*N* = 50,037) conducted in China showed that eating speed was positively associated with childhood central obesity. The fourth study (50) (*N* = 56,865) conducted in Japan showed that the risk of central obesity for the fast-eating group increased significantly with an OR of 1.97, and three studies (44, 46, 47) indicated that slow-eating speed was helpful in preventing central obesity.

Eating speed was divided into four groups in two studies (46, 47), both studies were conducted in Japan and were cross-sectional studies in adults. The two studies reported no significant association between eating speed and risk of central obesity in a moderate-eating group and a fast-eating group. However, one study (46) showed that the risk of central obesity for the very fast-eating group significantly increased. Another study (47) showed that the proportion of participants who had central obesity increased with increases in eating speed, irrespective of the status of glucose tolerance. Also, a cohort study (43) in Japanese middle-aged citizens (*N* = 141) showed that the decrease in WC from baseline to 1 year was greater in the intervention group (received health guidance to modify fast-eating speed) than in the control group (*P* < 0.05). A cross-sectional study (52) indicated that eating slower reduced WC for Japanese men (*N* = 59,717). A cross-sectional study (51) in Spanish adolescents (*N* = 1,978) also found that WC values were lower in those who reported an adequate speed of eating when compared with fast-eating speed.

Eleven studies (23, 24, 37, 39, 44–50) were included in the meta-analysis. Compared to the slow-eating speed subjects, subjects in the faster-eating group had a significantly higher risk of central obesity (OR = 1.54; 95% CI: 1.37–1.73; Figure 2). Additionally, in the subgroup analysis, subjects with very fast-eating speed had a more than triple risk of central obesity (OR = 3.14; 95% CI: 1.62–6.09), subjects in the fast-eating speed group also had a significantly higher risk (OR = 1.65, 95% CI: 1.31–2.07) (23, 37, 39, 55). The OR for the medium-eating speed group was 1.32 (95% CI: 1.15–1.52; Table 2) (23, 24, 37, 50).

### Elevated BP

A total of 11 studies examining the relationship between eating speed and elevated BP were identified and discussed in the systematic review (23, 24, 37, 39, 42, 43, 47, 50, 53–55). Ten studies involved adults and one study involved children (54). In addition, confounding factors of studies about elevated BP, including age, sex, BMI, and physical activity. Eating speed was divided into two groups in three studies (39, 42, 55), one study (55) (*N* = 11,195) conducted in Japan indicated that eating speed was significantly associated with a higher risk of elevated BP for men, but the risk did not reach statistical significance among women. However, the association did not reach statistical significance among both genders in another study (42). In addition, another study (39) also reported insignificant associations between eating speed and the risk of elevated BP. Eating speed was divided into three groups in four studies (23, 24, 37, 50), two studies (24, 50) regarded the moderate-eating group as the reference group. One study (50) showed that those who were in the slow-eating speed group had a significantly lower risk of elevated BP, and the OR of those who were in the fast-eating speed group was 1.2. Another study (24) also find statistical significance for the association between fast-eating speed and elevated BP. Two studies (23, 37) regarded the slow-eating group as the reference group, one study (23) conducted among adults in China found that compared with those who were in the slow-eating group, those who were in the moderate-eating group and the fast-eating group had a significantly higher risk for elevated BP; however, another study (37) did not find a significant association between eating speed and risk of increased BP. One study (47) divided eating speed into four groups, the study stratified participants into three subgroups according to the status of glucose tolerance (normal glucose/impaired fasting glucose/diabetes). The study found the risk of elevated BP for those who had normal glucose in the moderate-eating group was not significantly different, but those who had impaired fasting glucose in the relatively fast-eating group had a significantly higher risk compared with the slow-eating group. In the very fast-eating group, the risk for those who had impaired fasting glucose and diabetes significantly increased. A cross-sectional study (53) conducted in Korea found that the diastolic BP level among women was significantly different between each eating speed group (*P* < 0.05), a cohort study (54) among 1,490 children in Japan showed that eating speed was positively associated with systolic BP among boys. Another cohort study (43) among adults in Japan also found that systolic BP varied with the speed of eating (*P* < 0.01).

Six studies (23, 24, 37, 39, 50, 55) were included in the meta-analysis. As shown in Figure 2, subjects in the faster-eating group had a significantly higher risk of elevated BP compared with the slow-eating group (OR = 1.26, 95% CI: 1.13–1.40). Additionally, with subgroup analysis significantly higher risk of elevated BP was observed for the fast-eating speed group (OR = 1.25, 95% CI: 1.02–1.52) compared with the slow-eating group (23, 37, 39, 55). The OR for elevated BP for the medium-eating speed group was 1.30 (95% CI: 1.06–1.59; Table 2) (23, 24, 37, 50).

### Low HDL

Nine studies examining the relationship between eating speed and low HDL were included in our systematic review (23, 24, 37, 39, 43, 50, 53, 55, 56). Eight studies involved adults and one study involved children (56). Eating speed was divided into two groups in two studies (39, 55), a study (55) (*N* = 11,195) in Japan showed that the risk of low HDL for the fast-eating group increased only in men, but not in women. Another study (39) showed that compared with the non-fast-eating group, the risk of low HDL in the fast-eating group significantly increased.

Eating speed was divided into three groups in four studies (23, 24, 37, 50). Two studies (24, 50) used the moderate-eating group as the reference group. One study (50) showed that fast eating was associated with a higher risk of low HDL in both men and women, but slow eating was associated with a low risk of low HDL in men only. Another study (24) indicated an insignificant association between eating speed and low HDL.

Two studies (23, 37) used the slow-eating group as the reference group; one study (23) showed that both moderate eating and fast eating were associated with a higher risk of low HDL. Another study (37) showed that HDL-cholesterol level was significantly different between each eating speed group (*P* < 0.05). In addition, a cross-sectional study (53) conducted in Korea found that fast-eating speed was significantly associated with a higher risk of low HDL (*P* < 0.05). A 12-month RCT (56) involving retraining eating behavior (such as, reducing eating speed) by a computerized device (Mandometer) showed that compared with the standard care group, HDL-cholesterol concentration of obese children for the Mandometer group improved significantly (*P* < 0.05). However, this controlled, non-randomized, intervention study (43) that analyzed 141 participants with MetS at baseline showed that the risk of low HDL for participants in the intervention group was not significantly lower than that in the control group (*P* = 0.242).

As shown in Figure 2, six studies (23, 24, 37, 39, 50, 55) were included in the meta-analysis, the results indicated that the risk of low HDL in the faster-eating group increased compared with the slow eating group (OR = 1.23, 95% CI: 1.15–1.31). The risk of low HDL for the fast-eating speed group was 29% higher than the slow-eating group (OR = 1.29, 95% CI: 1.17–1.42) (23, 37, 39, 55). Additionally, in subgroup analysis, the risk of low HDL of the medium-eating speed group was 17% higher than the slow-eating group (OR = 1.17, 95% CI: 1.07–1.29; Table 2) (23, 24, 37, 50).

### Elevated TG

Eight studies examining the relationship between eating speed and elevated TG were discussed in the systematic review (23, 24, 37, 39, 43, 50, 53, 55). These studies involved only adults. In addition, confounding factors of studies about elevated TG included age, sex, BMI, physical activity, and total energy intake, etc. Eating speed was divided into two groups in two studies (39, 55). Although no significant associations were found between eating speed and elevated TG in the cohort study (39) among adults in Japan and China, the study (55) conducted in Japan showed that the risk of elevated TG for the fast-eating speed group was significantly higher compared with the slow-eating speed group both in men and women.

Eating speed was divided into three groups in three studies (23, 37, 50), two studies (23, 37) used slow-eating group as the reference group, and one study (23) indicated that eating speed was associated with a higher risk for elevated TG in males. No significant association was found between moderate-eating speed and elevated TG in another study (37), but fast-eating was significantly associated with a higher risk of elevated TG. Two studies (24, 50) regarded the moderate-eating group as the reference group; one study (50) showed that the association between slow eating and lower odds of elevated TG was statistically significant; the risk of the presence of elevated TG was positively associated with fast eating. However, no significant associations were found between eating speed and elevated TG in another study (24). In addition, one study (53) conducted in Korea showed that elevated TG levels between each group were significantly different (*P* < 0.05). The cohort study (43) conducted in Japan showed that the presence of elevated TG was significantly different in different eating speed groups (*P* = 0.002).

Six studies (23, 24, 37, 39, 50, 55) were included in the meta-analysis. The results showed that the risk of elevated TG for the faster-eating-speed group was higher compared with the slow-eating speed group (OR = 1.29, 95% CI: 1.18–1.42; Figure 2). In subgroup analysis, the risk of elevated TG for the fast-eating speed group was significantly higher than the slow-eating group with an OR of 1.38 (95% CI: 1.22–1.56) (23, 37, 39, 55), and the OR for the medium-eating speed group was 1.34 (95% CI: 1.12–1.61; Table 2) (23, 24, 37, 50).

### Elevated FPG

Twelve studies examining the relationship between eating speed and elevated FPG were discussed in the systematic review (23, 24, 37, 39, 42, 50, 53, 55, 57–60). These 12 studies were all conducted in adults. In addition, confounding factors of studies about elevated FPG included age, sex, BMI, physical activity, total energy intake, etc. Eating speed was divided into two groups in three studies (39, 55, 59). Two studies (55, 59) conducted in Japan indicated statistically significant differences of risk of elevated FPG between fast-eating and slow-eating group, one study (59) showed that the risk of elevated FPG for fast-eating speed was 1.10-fold (95% CI: 1.05–1.17), another study (55) showed that the risk of elevated FPG for fast-eating speed was statistically significant for men. However, two studies (39, 42) conducted in Japan indicated an insignificant association between eating speed and elevated FPG.

Eating speed was divided into three groups in six studies (23, 24, 37, 50, 58, 60). Four studies (23, 37, 58, 60) used the slow-eating group as the reference group, one study (23) indicated that eating speed was associated with a higher risk for elevated TG in males, one study (58) indicated that modified eating speed could be an acceptable health intervention for the prevention of type 2 diabetes. In addition, a case-control study (60) indicated that fast-eating speed increased the risk of type 2 diabetes mellitus significantly. However, no significant association was found between moderate-eating speed and elevated FPG in another study (37). Two studies (24, 50) regarded the moderate-eating group as the reference group, one study (50) showed that the association between slow eating and lower odds of elevated TG was statistically significant, fast eating was associated with a higher risk of elevated FPG. However, no significant associations were found between eating speed and elevated FPG in another study (24). In addition, one study (53) conducted in Korea showed that the difference of elevated FPG between each group was statistically significant. One study (57) showed that eating fast was associated with insulin resistance in middle-aged Japanese.

As shown in Figure 2, nine studies (23, 24, 37, 39, 50, 55, 58–60) were included in the meta-analysis, the results showed that fast-eating speed increased the risk of elevated FPG (OR = 1.16; 95% CI: 1.06–1.27). Compared with the slow-eating group, the risk of elevation for FPG in the fast-eating speed group increased significantly (OR = 1.20; 95% CI: 1.06–1.36) (23, 37, 39, 55, 58–60). However, the risk was not significantly different for the medium-eating speed group (OR = 1.14, 95% CI: 0.99–1.31) compared with the slow-eating group (23, 24, 37, 50, 58, 60) (Table 2).

### Subgroup Analysis and Meta Regression Analysis

Results of subgroup analysis showed that there was a high level of heterogeneity in studies of eating speed with MetS, central obesity, elevated BP, a moderate level of heterogeneity for studies with elevated FPG and elevated TG, and a low level of heterogeneity for low HDL. The heterogeneity of the subgroup analysis stratified by the eating speed did not change significantly. However, in the subgroup analysis stratified by age, gender, location, eating speed categorization, and study design, the heterogeneity decreased substantially. The results of meta regression analysis suggested that the heterogeneity between studies may be due to differences in gender and eating speed categorization. The results above indicate that age, gender, study location, and study design may be possible sources for the high level of heterogeneity among studies between eating speed and the risk of elevated BP, central obesity, or MetS (Table 2, Supplementary Table 6).

## Discussion

To the best of our knowledge, this is the first systematic review and meta-analysis to assess the relationship between eating speed with risk of MetS and its components. Our study included cohort studies, cross-sectional studies, case-control studies, and RCTs. The results suggest that the risk of MetS and its components increased significantly as eating speed increased, especially in the fast and very fast-eating speed group. In addition, the risk of MetS and its components was significantly associated with the medium-eating speed except for FPG. Our findings indicate the potential importance of interventions to minimize the excessive speed of eating to prevent the related adverse MetS outcomes.

For the medium-eating speed group, the CI was significant for risk of MetS, central obesity, elevated BP, elevated TG, and low HDL was significant, but not significant for elevated FPG. There are two possible hypotheses to explain this phenomenon. First, for the association between eating speed and risk of elevated FPG, the association in male participants is much more profound than in females. For the subgroup analysis of the association between medium eating and FPG, most included studies (23, 24, 37, 50, 58, 60) involved much more males with an average ratio of male of 75.78%, which is higher than other studies of meta-analysis for other outcomes. However, male participants are most fast eaters (59), medium-eating speed is not as common as in females, which may lead to relatively smaller sample size and then lower statistical power to test the significant associations in the medium-speed group. The second hypothesis is that the association between eating speed and risk of elevated FPG, the threshold value of eating speed to increase the risk of elevated FPG might be higher than other outcomes. These hypotheses should be further explored or tested in future studies.

A previous intervention study showed that health guidance about MetS and eating quickly decreased the prevalence of MetS, BMI, WC, and TG (43). Various studies have also confirmed the effectiveness of dietary interventions for individuals with MetS, among these studies, individuals with MetS had significantly lower body weight, WC, and TG levels after dietary intervention (55, 58, 61, 62). Previous studies have shown that eating quickly was associated with a higher risk of type 2 diabetes mellitus in middle-aged men in Japan (44, 59), and those who ate quickly had a higher risk (OR = 1.5) of insulin resistance (HOMA-IR ≥ 2.0) (63). Additionally, some studies have demonstrated that a fast-eating speed can lead to higher levels of serum insulin resistance (64), increased FPG levels, and a higher risk of MetS. Fast-eating speed can also cause overeating, resulting in higher postprandial blood glucose levels (45, 64–66). Eating slowly can decrease energy intake, leading to satiety or improvements in insulin sensitivity (67), these findings support our results. A previous study showed that a lower calorie diet can improve the levels of HDL and other lipids (67). Eating too quickly makes it difficult for the body to recognize calories and satiety, resulting in excessive calorie intake (68). As a result, fast-eating speed can affect HDL levels by influencing a greater energy intake. Therefore, it can be speculated that controlling the speed of eating might reduce energy intake and prevent the development of low HDL, which is consistent with our findings. Excessive calorie intake can also lead to an increased risk of central obesity; a prospective study confirmed that eating slowly is beneficial for preventing obesity in non-overweight/obese girls (67). Several RCTs showed that WC was lower in an intervention group (adjusting dietary intake) at the end of the dietary program (69), and modifications in eating speed was efficient, cost-effective means for weight management for centrally obese or healthy adolescents (67, 70). A recent observational study indicated that modification in eating speed can reduce the risk of obesity in patients with type 2 diabetes (63). The study suggested that slowing down eating speed may be beneficial in preventing obesity. Additionally, one study showed that those who ate quickly have higher levels of interleukin-1β and interleukin-6 compared with those who ate slowly, even after taking energy intake and BMI into account (58). The increased cytokine activity could lead to high BP levels via increases in renal sodium and water retention (64, 66). This may also influence the activity of plasma noradrenaline and the sympathetic nervous system, and our results are consistent with the findings of these studies.

Our study has significant strengths. First, this is the first meta-analysis and systematic review exploring the associations between eating speed with the risk of MetS and its components. Second, although most of the results showed significant heterogeneity, the quality of included studies was in the medium-to-high range. Third, the sample size of included studies was relatively large, our meta-analysis with a large sample size provides relatively strong statistical power to test the associations. Fourth, the study involved 465,155 subjects from different countries.

However, there are also several limitations. The results indicated a high heterogeneity between studies. Several reasons may explain the high level of heterogeneity. First, assessment methods for eating speed varied between studies, among the 29 included articles, a FFQ was used in four articles, the diet history questionnaire (DHQ) was used in two articles, self-administered questionnaire was used in other articles. FFQ, DHQ, and other self-administered questionnaires can introduce possible recall bias, leading to different classification or misclassification of eating speed. Additionally, measurement error and different measurement methods for dietary intake could also introduce misclassification. Although we conducted meta-regression analysis to estimate the heterogeneity and explore the source of heterogeneity, actually for each meta-regression model, the number of included studies was limited. If future relevant studies were conducted using wearable cameras to accurately estimate the speed of eating, it would more informative and precise to verify the associations and demonstrate the source of heterogeneity. Second, definitions of MetS and its components in the included studies were different in some studies. There are several definitions for MetS outlined by different expert groups. As a result, it is difficult to compare risk factors or prevalence for MetS across all studies. Among included studies, most studies have adopted the ATP III NCEP definition. However, it has been confirmed that current ATP III NCEP definitions have resulted in an unrealistic low prevalence of MetS in Asian cohorts, suggesting that ethnic-specific cutoff values are needed urgently (68, 71). Third, most of the included studies were conducted in Asian countries, especially Japan, China, and Korea. Only four studies were conducted in non-Asian countries (37, 51, 56, 60)—two in Spain, one in England, one in America. Fourth, among the included studies, only five studies were conducted in children (44, 48, 51, 54, 56), most studies were conducted in adults. This may also explain in part the high heterogeneity observed.

## Conclusion

Epidemiological data have demonstrated significant associations between eating speed with the risk of Mets and its components. This suggests that there is a significant contribution from fast eating in the development of metabolic disorders (57). Eating slowly might be a protective factor for central obesity, whereas eating fast is likely to be a risk factor for Mets, central obesity, elevated BP, low HDL, elevated TG, and elevated FPG. Future recommendations should aim to control the speed of eating to prevent MetS and its components. Moderation in eating speed may be an important and relatively simple measure to prevent MetS and its related metabolic disorders (47). It is also important to unify the measurement and classification of eating speed, similarly, ethnic-specific cutoff values for eating speed are an essential further scientific and research direction.

## Data Availability Statement

The original contributions presented in the study are included in the article/Supplementary Material, further inquiries can be directed to the corresponding author/s.

## Author Contributions

S-QY, Y-DY, and Y-ML designed and conducted the research, collected and analyzed the data, and wrote the paper. YZ, Y-YL, Q-YH, Y-DY, BQ, and JB helped with the data interpretation, contributed to the discussion, and revised the paper. S-QY had primary responsibility for the final content of the manuscript. All authors participated in critically revising and approving the final manuscript.

## Funding

This study was supported by the National Natural Science Foundation of China (no. 81903336), the Hunan Provincial Natural Science Foundation of China (no. 2019JJ50376), the Scientific Research Project of Hunan Health Committee (no. 202112031516), and the Open Project for Postgraduates of Hunan Normal University (no. KF2021036). The funders had no role in the design, analysis, or writing of this article.

## Conflict of Interest

The authors declare that the research was conducted in the absence of any commercial or financial relationships that could be construed as a potential conflict of interest.

## Publisher's Note

All claims expressed in this article are solely those of the authors and do not necessarily represent those of their affiliated organizations, or those of the publisher, the editors and the reviewers. Any product that may be evaluated in this article, or claim that may be made by its manufacturer, is not guaranteed or endorsed by the publisher.

## Supplementary Material

Supplementary File 1 The search strategy of the present systematic review and meta-analysis.

Supplementary Table 1 The detailed characteristics of the studies reporting on the association between eating speed and metabolic syndrome and its components.

Supplementary Table 2 Assessment of the quality of 20 cross-sectional studies by the Agency for Healthcare Research and Quality (AHRQ) scale.

Supplementary Table 3 Assessment of the quality of seven cohort studies and one case-control study by Newcastle-Ottawa Scale (NOS).

Supplementary Table 4 Assessment of the quality of one randomized controlled trial by Cochrane Scale.

Supplementary Table 5 Begg's test and Egger's test of the included studies in the meta-analysis.

Supplementary Table 6 Meta-regression models of multivariable-adjusted analyses.

Supplementary Figure 1 Begg's funnel plots of the included studies in the meta-analysis. (Only when the number of included studies was more than five, the Begg's test and plot were conducted. MetS, metabolic syndrome; BP, blood pressure; FPG, fasting plasma glucose; TG, triglycerides; HDL, high-density lipoprotein).

Supplementary Figure 2 Sensitivity analysis of the included studies in the meta-analysis. (The slow eating group was used as the reference group. MetS, metabolic syndrome; BP, blood pressure; FPG, fasting plasma glucose; TG, triglycerides; HDL, high-density lipoprotein).
