# Supplementary material for: Association Between Eating Speed and Metabolic Syndrome: A Systematic Review and Meta-Analysis
Source: Front Nutr. 2021 Oct 20;8:700936. doi: 10.3389/fnut.2021.700936 (PMC8564065; doi:10.3389/fnut.2021.700936)
Supplement: Supplementary File 1 — The search strategy of the present systematic review and meta-analysis. [file Data_Sheet_1.doc]

**Supplementary File 1**

**The search strategy of the present systematic review and meta-analysis**

1. Web of Science (The number of recorded articles:1767)

1.'rapid':all

2.'fast':all

3.'quick':all

4.'quickly':all

5.'speed':all

6.'rate':all

7.'slow':all

8.'time':all

9. 'duration':all

10.1 or 2 or 3 or 4 or 5 or 6 or 7 or 8 or 9

11.'meal':all

12.'eat':all

13.'eater':all

14.11 or 12 or 13

15.'metabolic syndrome':all

16.'central obesity':all

17.'abdominal obesity':all

18.'high density lipoprotein':all

19.'blood pressure':all

20.'sbp':all

21.'dbp':all

22.'hypertension':all

23.'triglyceride':all

24.'hdl':all

25.'fpg':all

26.15 or 16 or 17 or 18 or 19 or 20 or 21 or 22 or 23 or 24 or 25

27.'randomized controlled trial':all

28.'RCT':all

29.'cohort':all

30.'cross-sectional ':all

31.'case-control ':all

32.27 or 28 or 29 or 30 or 31

33.10 and 14 and 26 and 32

[all=all fields]

1. Medline (The number of recorded articles:1912)

1.'rapid':TS

2.'fast':TS

3.'quick':TS

4.'quickly':TS

5.'speed':TS

6.'rate':TS

7.'slow':TS

8.'time':TS

9.'duration':TS

10.1 or 2 or 3 or 4 or 5 or 6 or 7 or 8 or 9

11.'meal':TS

12.'eat':TS

13.'eater':TS

14.11 or 12 or 13

15.'metabolic syndrome':TS

16.'central obesity':TS

17.'abdominal obesity':TS

18.'high density lipoprotein':TS

19.'blood pressure':TS

20.'sbp':TS

21.'dbp':TS

22.'hypertension':TS

23.'triglyceride':TS

24.'hdl':TS

25.'fpg':TS

26.15 or 16 or 17 or 18 or 19 or 20 or 21 or 22 or 23 or 24 or 25

27.'randomized controlled trial':TS

28.'RCT':TS

29.'cohort':TS

30.'cross-sectional ':TS

31.'case-control ':TS

32.27 or 28 or 29 or 30 or 31

33.10 and 14 and 26 and 32

[TS= topic]

1. PubMed (The number of recorded articles:3023)

1.'rapid':all

2.'fast':all

3.'quick':all

4.'quickly':all

5.'speed':all

6.'rate':all

7.'slow':all

8.'time':all

9. 'duration':all

10.1 or 2 or 3 or 4 or 5 or 6 or 7 or 8 or 9

11.'meal':all

12.'eat':all

13.'eater':all

14.11 or 12 or 13

15.'metabolic syndrome':all

16.'central obesity':all

17.'abdominal obesity':all

18.'high density lipoprotein':all

19.'blood pressure':all

20.'sbp':all

21.'dbp':all

22.'hypertension':all

23.'triglyceride':all

24.'hdl':all

25.'fpg':all

26.15 or 16 or 17 or 18 or 19 or 20 or 21 or 22 or 23 or 24 or 25

27.'randomized controlled trial':all

28.'RCT':all

29.'cohort':all

30.'cross-sectional ':all

31.'case-control ':all

32.27 or 28 or 29 or 30 or 31

33.Filter:Human

34.10 and 14 and 26 and 32 and 33

[all=all fields]

1. EMBASE (The number of recorded articles:1798)

1.'rapid':all

2.'fast':all

3.'quick':all

4.'quickly':all

5.'speed':all

6.'rate':all

7.'slow':all

8.'time':all

9. 'duration':all

10.1 or 2 or 3 or 4 or 5 or 6 or 7 or 8 or 9

11.'meal':all

12.'eat':all

13.'eater':all

14.11 or 12 or 13

15.'metabolic syndrome':all

16.'central obesity':all

17.'abdominal obesity':all

18.'high density lipoprotein':all

19.'blood pressure':all

20.'sbp':all

21.'dbp':all

22.'hypertension':all

23.'triglyceride':all

24.'hdl':all

25.'fpg':all

26.15 or 16 or 17 or 18 or 19 or 20 or 21 or 22 or 23 or 24 or 25

27.'randomized controlled trial':all

28.'RCT':all

29.'cohort':all

30.'cross-sectional ':all

31.'case-control ':all

32.27 or 28 or 29 or 30 or 31

33.10 and 14 and 26 and 32

[all=all fields]
