# Supplementary material for: Association Between Eating Speed and Metabolic Syndrome: A Systematic Review and Meta-Analysis
Source: Front Nutr. 2021 Oct 20;8:700936. doi: 10.3389/fnut.2021.700936 (PMC8564065; doi:10.3389/fnut.2021.700936)
Supplement: Supplementary Table 1 — The detailed characteristics of the studies reporting on the association between eating speed and metabolic syndrome and its components. [file Data_Sheet_2.pdf]

frontiers

Supplementary Material

Association between eating speed with metabolic syndrome and its components: a systematic review and meta-analysis

Shu-qian Yuan[1], Ying-ming Liu[1], Wei Liang[2,3], Fei-fei Li[2,3], Yuan Zeng[1], Yin-yue Liu[1], Shu-zhen Huang[1], Quan-yuan He[1], Binh Quach[3], Jiao Jiao[4], Julien S. Baker[2,3]*, Yi-de Yang[1]*

[1]Key Laboratory of Molecular Epidemiology of Hunan Province, School of Medicine, Hunan Normal University, Changsha, 410006, China

[2] Centre for Health and Exercise Science Research, Hong Kong Baptist University, Kowloon Tong 999077, Hong Kong, China;

[3] Department of Sport, Physical Education and Health, Hong Kong Baptist University, Kowloon Tong 999077, Hong Kong, China;

[4] Dr. Stephen Hui Research Centre for Physical Recreation and Wellness, Hong Kong Baptist University, Kowloon Tong 999077, Hong Kong, China.

***Correspondence:**

Yi-de Yang

yangyide2007@126.com

Julien S Baker

jsbaker@hkbu.edu.hk

Contents:

Table S1. The detailed characteristics of the studies reporting on the association between eating speed and metabolic syndrome and its components.

Table S2. Assessment of the quality of 20 cross-sectional studies the Agency for Healthcare Research and Quality (AHRQ) scale.

Table S3. Assessment of the quality of 7 cohort studies and 1 case-control study by Newcastle-Ottawa Scale (NOS).

Table S4. Assessment of the quality of 1 randomised controlled trial by Cochrane Scale.

Table S5. Begg's test and Egger's test of the included studies in the meta-analysis.

Figure S1. Begg's funnel plots of the included studies in the meta-analysis. (Only when the number of included studies was more than 5, the Begg's test and plot were conducted. MetS, metabolic syndrome; BP, blood pressure; FPG, fasting plasma glucose; TG, triglycerides; HDL, high-density lipoprotein)

Figure S2. Sensitivity analysis of the included studies in the meta-analysis. (The slow eating group was used as the reference group. MetS, metabolic syndrome; BP, blood pressure; FPG, fasting plasma glucose; TG, triglycerides; HDL, high-density lipoprotein)

Table S6. Meta-regression models of multivariable adjusted analyses

Table S1. The detailed characteristics of the studies reporting on the association between eating speed and metabolic syndrome and its components

Reference	Study design	Country	sample size (% men)	Age（years）	population	Evaluation of eating rate	Category of eating rate		Outcome	OR/P
							Quickly	Reference		
Pey Sze Teo, et al[45]	Cross-sectional study	Singapore	7011(44.8)	49.8±13.0	adults	Self-report	Medium/Fast	Slow	Abdominal overweight	Medium: OR=1.40(1.18-1.67)<br>Fast: OR=1.84(1.52-2.21)
Lixin Tao, et al[23]	Cross-sectional study	China	7972(88.26)	29-48	adults	Self-report	Medium/Fast	Slow	MetS; central obesity;<br>BP; FPG;TG;<br>HDL	Medium: OR=1.65(1.32-2.07)<br>OR=1.39(1.12-1.72)<br>OR=1.57(1.28-1.92)<br>OR=1.11(0.88-1.41)<br>OR=1.25(1.00-1.55)<br>OR=1.22(1.02-1.45)<br>Fast: OR=2.27(1.80-2.86)<br>OR=1.68(1.35-2.09)<br>OR=1.82(1.48-2.24)<br>OR=1.18(0.93-1.51)<br>OR=1.51(1.21-1.88)<br>OR=1.33(1.11-1.60)
Chikanobu Sonoda, et al[46]	Cross-sectional study	Japan	863(100)	34-59	adults	Self-report	Ordinary/Fast/Very fast	Slow, very slow	WC≥85cm,<br>WC≥90cm,	Ordinary: OR=1.72 (0.96–3.11)<br>OR=2.34 (1.08–5.05)<br>Fast: OR=3.13 (1.74–5.64)<br>OR=3.90 (1.82–8.34)<br>Very fast: OR=6.59 (2.37–18.48)<br>OR=5.22 (1.81–15.06)
AESUN SHIN, et al[35]	Cross-sectional study	Korea	7081(100)	≥30	adults	Self-report	Average/Fast	Slow	MetS	Average: OR= 1.66 (1.19-2.32)<br>Fast: OR= 2.23 (1.60-3.12)
Xia Zeng, et al[48]	Cross-sectional study	China	50037(51.1)	11.28±3.07	children	Self-report	Slow/Fast	Medium	Abdominal obesity, WHtR≥0.5	Slow: OR=0.70 (0.65,0.75)<br>OR=0.71 (0.65,0.78)<br>Fast: OR=1.51 (1.41,1.61)<br>OR=1.54 (1.43,1.65)
Rei Otsuka, et al[57]	Cross-sectional study	Japan	3465(78.1)	35-69	adults	Self-report	Very slow/Relatively slow /Relatively Fast/Very fast	Medium	Insulin resistance (HOMA-IR)	*P*<0.01
Hirotaka Ochiai, et al[44]	Cross-sectional study	Japan	2136(50.3)	12-13	children	Self-report	Eating quickly	Not Eating quickly	WHtR ≥ 0.5	Boys: Eating quickly: OR=2.05 (1.31–3.23)<br>Girls: Eating quickly: OR=2.09 (1.15–3.81)
K.S. Lee, et al[53]	Cross-sectional study	Korea	8775(54.9)	20-80	adults	Self-report	10≤and <15 min/<br>5≤and <10 min/<5 min	NA	Low HDL-C, High TG,<br>High blood glucose,<br>High blood pressure	*P*<0.05
Shiun Dong Hsieh, et al[55]	Cross-sectional study	Japan	11195(74.0)	men:51.6±9.4<br>women:52.9±10.1	adults	Self-report	Eating rapidly	Not eating until feeling full and not eating rapidly	Hypertension,<br>Hyperglycaemia,<br>Hypertriglyceridemia,<br>Low HDL-C	Men: OR= 1.36(1.22–1.51)<br>OR= 1.29(1.16–1.43)<br>OR= 1.43(1.27–1.61)<br>OR= 1.30(1.10–1.54)<br>Women: OR= 1.16(0.93–1.44)<br>OR= 1.37(1.07–1.73)<br>OR= 1.59(1.13–2.22)<br>OR= 1.26(0.95–1.68)
Alihiro Kudo, et al[59]	Cohort study	Japan	197825(38.0)	64	adults	Self-report	Fast	Slow	Diabetes	OR=1.10(1.05-1.17)
Kazumasa Yamagishi, et al[54]	Cohort Study	Japan	1490(42.8)	6-12	children	Self-report	Never/ Quit/ Newly/ Continuous	NA	SBP	P=0.01
Mizuki Saito, et al[36]	Cross-sectional study	Japan	2379(40.35)	75-80	adults	Self-report	Normal/Fast	Slow	MetS	Normal: OR=1.34(0.92-1.95)<br>Fast: OR=2.06(1.35-3.16)
Parvane Saneei, et al[49]	Cross-sectional study	Iranian	7958(NA)	NA	adults	Self-report	Moderate to slow/<br>Moderate to fast	Moderate	WC ≥80 cm for women and ≥94 cm for men<br>WC ≥88 cm for women and ≥102 cm for men	Moderate to slow: OR=0.87 (0.73–1.05)<br>OR=0.86 (0.70–1.06)<br>Moderate to fast: OR=1.19 (0.95–1.49)<br>OR=1.07 (0.83–1.37)
Saleem Perwaiz Iqbal, et al[38]	Cross-sectional study	Malaysia	481(35.1)	≥18	adults	Self-report	Fast	Not Fast	Metabolic syndrome	OR=1.15 (0.75–1.78)
Satsue Nagahama, et al[50]	Cross-sectional study	Japan	56865(73.54)	17-99	adults	Self-report	Slow/Fast	Normal	Central obesity, High blood pressure, High fasting plasma glucose, High triglyceride, Low HDL-C	Men:<br>Slow: OR=0.63 (0.56-0.70)<br>OR=0.74 (0.68-0.81)<br>OR=0.78 (0.71-0.87)<br>OR=0.90 (0.82-0.98)<br>OR=0.83 (0.73-0.96)<br>Fast: OR=1.97 (1.88-2.07)<br>OR=1.20 (1.15-1.26)<br>OR=1.16 (1.11-1.22)<br>OR=1.33 (1.27-1.39)<br>OR=1.36 (1.28-1.45)<br>Women:<br>Slow: OR=0.73 (0.64-0.83)<br>OR=0.76 (0.65-0.88)<br>OR=1.03 (0.85-1.25)<br>OR=0.81 (0.66-1.00)<br>OR=0.89 (0.74-1.08)<br>Fast: OR=1.44 (1.33-1.56)<br>OR=1.10 (1.15-1.20)<br>OR=1.16 (1.03-1.31)<br>OR=1.13 (1.01-1.27)<br>OR=1.12(1.00-1.26)
Masaru Sakurai, et al[58]	Cross-sectional study	Japan	2050(100)	45.9±6.0	adults	Self-report	Medium/Fast	Slow	type 2 diabetes	Medium: OR=1.68 (0.93 - 3.02)<br>Fast: OR=1.97 (1.10 - 3.55)

Bing Zhu, et al[39]	Cohort Study	Japan	8941(38.3)	63.7 ±7.9	adults	Self-report	Fast	Not fast	metabolic syndrome<br>WC, HDL-C, TG, BP, BG	Fast: OR=1.3(1.05-1.60)<br>OR=1.35(1.10-1.66)<br>OR=1.37(1.12-1.67)<br>OR=1.15(0.99-1.31)<br>OR=1.10(0.94-1.27)<br>OR=0.93(0.75-1.16)
Indira Paz-Graniel, et al[37]	Cross-sectional study	Spain	792(42.93)	50-80	adults	Self-report	Medium/Fast	Slow	MetS, Central obesity,<br>Hypertriglyceridemia,<br>Low HDL-C, High Blood Pressure,<br>High fasting glucose	Fast: OR=0.99(0.86-1.14) Medium: OR=1.02 (0.88–1.18)<br>OR=0.95(0.87-1.04) OR=1.00 (0.91–1.10)<br>OR=1.47(1.08-2.02) OR=1.32 (0.95–1.85)<br>OR=0.94(0.68-1.30) OR=0.96 (0.68–1.37<br>OR=1.00(0.97-1.04) OR=1.00 (0.96–1.03)<br>OR=0.92(0.80-1.06) OR=1.00 (0.87–1.15)
Daisuke Ekuni, et al[43]	Cohort Study	Japan	141(53.19)	65.0 ±6.7	adults	Self-report	Intervention	Control	WC, SBP, DBP, Triglyceride, High-density lipoprotein cholesterol, Low-density lipoprotein cholesterol	Waist circumference: P<0.001<br>Systolic blood pressure: P<0.001<br>Diastolic blood pressure: P=0.942<br>Triglyceride: P=0.002<br>High-density lipoprotein cholesterol: P=0.242<br>Low-density lipoprotein cholesterol: P=0.195
Akiko Nanri, et al[24]	Cohort Study	Japan	1018(88.41)	19-68	adults	Self-report	Slow/Fast	Medium	MetS, Central obesity<br>High blood pressure,<br>High fasting plasma glucose,<br>High triglyceride, Low HDL-C	Slow: OR=0.93(0.34-2.53) Fast; OR=2.13 (1.23–3.68)<br>OR=0.65(0.37-1.13) OR=1.64 (1.22–2.20)<br>OR=0.70(0.45-1.10) OR=1.22 (0.93–1.59)<br>OR=0.93(0.51-1.68) OR=1.24 (0.87–1.76)<br>OR=0.68(0.38-1.23) OR=1.31 (0.95–1.81)<br>OR=0.52(0.27-1.01) OR=0.96 (0.67–1.37)
T. Ohkuma, et al[47]	Cross-sectional study	Japan	7275(51.39)	≥40	adults	Self-report	Medium/Relatively fast/Very fast	Slow	Elevated waist circumference<br>Elevated blood pressure	Normal glucose IFG Diabetes<br>Medium: OR=1.44 (1.07, 1.95) OR=1.97 (1.33, 2.92) OR=0.89 ([illegible]<br>OR=1.01 (0.74, 1.37) OR=1.28 (0.86, 1.90) OR=1.17 ([illegible]<br>Relatively fast:<br>OR=2.30 (1.62, 3.27) OR=1.84 (1.19, 2.86) OR=1.06 ([illegible]<br>OR=1.31 (0.93, 1.86) OR=1.60 (1.02, 2.51) OR=1.07 ([illegible]<br>Very fast:<br>OR=3.31 (1.90, 5.76) OR=4.10 (2.29, 7.34) OR=1.55 ([illegible]<br>OR=0.79 (0.46, 1.35) OR=2.32 (1.24, 4.34) OR=1.56 ([illegible]
Ford AL, et al[56]	Randomised controlled trial	England	106(NA)	9-18	children	by a computerised device	Mandometer group	Standard care group	HDL-C	*P*<0.05
Tajima Miki, et al[22]	Cohort Study	Japan	3137(56.80)	47.5 ±9.1	adults	Self-report	Slow	Others	MetS	OR=0.49 (0.24-0.996)
Radzevičienė, Lina, et al[60]	A case-control study	America	234(39.28)	34-86	adults	Self-report	The same/Faster	Slow	Type 2 diabetes	OR=2.52 (1.56-4.06)
Hurst Yumi, et al[52]	Cross-sectional study	Japan	59717(100)	40-69	adults	Self-report	Slow/normal	Faster	WC	Slow: OR= 0.58 (0.54 -0.63)<br>Normal: OR=0.71 (0.68 -0.75)
Sonia GÔmez-MartÎnez, et al[51]	Cross-sectional study	Spain	1978(48.58)	13-18.5	adolescents	Self-report	Quick	adequate	WC	Boy: *P*<0.001<br>Girl: *P*=0.002
Youji Sogai, et al[40]	Cross-sectional study	Japan	4,912(20.72)	30-39	adults	Self-report	Fast	Not fast	MetS	Men: OR=1.65(1.25-2.18)<br>Women:OR=2.07(1.50-2.85)
Eiichi Ashizawa, et al[42]	Cohort Study	Japan	8316(74.13)	20-64	adults	Self-report	After the intervention	Before the intervention	MetS<br>High blood pressure<br>diabetes	Men: OR=0.84(0.70-1.00)<br>OR=0.98(0.84-1.20)<br>OR=0.76(0.59-0.96)<br>Women:OR=1.5(0.88-2.40)<br>OR=0.8(0.56-1.10)<br>OR=0.71(0.36-1.4)
Joe, Heegyung, et al[41]	Cross-sectional study	Korea	1005(0.00)	20-49	adults	Self-report	Fast	Not fast	MetS	*P*=0.017

MetS, metabolic syndrome; BP, blood pressure; SBP, systolic blood pressure; DBP, Diastolic blood pressure; BG, blood glucose; FPG, fasting plasma glucose; TG, triglycerides; HDL, high-density lipoprotein; IFG, impaired fasting glucose; WC, waist circumference; WHt[illegible] periodontal index.

Table S2. Assessment of the quality of 20 cross-sectional studies the Agency for Healthcare Research and Quality (AHRQ) scale.

**Study**	**1**	**2**	**3**	**4**	**5**	**6**	**7**	**8**	**9**	**10**	**11**	**Total points**	**Quality evaluation**
Indira Paz-Graniel, et al[37]	1	0	1	0	1	0	1	0	1	1	1	7	moderate
Pey Sze Teo, et al [45]	1	0	1	1	0	1	1	0	1	1	1	8	high
Lixin Tao, et al[23]	1	0	1	0	0	1	1	1	1	1	1	8	high
Chikanobu Sonoda, et al[46]	1	1	1	1	0	0	0	1	0	0	1	6	moderate
AESUN SHIN, et al[35]	1	0	1	0	0	1	1	1	1	1	1	8	high
Xia Zeng, et al[48]	1	0	1	1	0	0	0	1	0	0	1	5	moderate
Rei Otsuka, et al[57]	1	0	1	0	0	1	1	1	0	1	1	7	moderate
Hirotaka Ochiai, et al[44]	1	0	1	0	0	1	1	1	0	1	1	7	moderate
K.S. Lee, et al[53]	1	0	1	0	0	1	1	0	0	1	1	6	moderate
Shiun Dong Hsieh, et al[55]	1	0	0	1	0	1	0	0	0	0	1	4	moderate
Mizuki Saito, et al[36]	1	0	1	0	0	1	0	1	0	0	1	5	moderate
T. Ohkuma, et al[47]	1	0	1	0	0	1	1	1	0	1	1	7	moderate
Parvane Saneei, et al[49]	1	1	1	0	0	1	0	1	0	1	1	6	moderate
Saleem Perwaiz Iqbal, et al[38]	1	1	1	0	0	1	1	1	0	0	1	7	moderate
Hurst Yumi, et al[52]	1	1	1	0	0	1	1	1	0	0	1	7	moderate
Satsue Nagahama, et al[50]	1	0	1	0	0	1	1	1	1	0	1	7	moderate
Masaru Sakurai, et al[58]	1	0	1	0	0	1	0	1	0	0	1	5	moderate
Sonia GÔmez-MartÎnez, et al[51]	1	0	1	0	0	1	1	0	1	0	1	6	moderate
Youji Sogai, et al[40]	1	1	1	0	0	0	1	0	0	1	0	5	moderate
Joe, Heegyung, et al[41]	1	1	1	0	0	0	1	1	1	1	0	7	moderate

Table S3. Assessment of the quality of 7 cohort studies and 1 case-control study by Newcastle-Ottawa Scale (NOS).

	Selection				Comparability	Outcomes				
Cohort study	representativeness of the cohort	Selection of the non-exposed cohort	ascertainment of exposure	demonstration that outcome of interest was not present at start of study	comparability	assessment of outcome	follow-up long enough for outcomes to occur	adequacy of follow up of cohorts	total	quality evaluation
Alihiro Kudo, et al[59]	1	1	1	1	1	1	1	+1	8	high
Kazumasa Yamagishi, et al[54]	1	1	1	-	1	1	1	-	6	moderate
Bing Zhu, et al[39]	1	1	1	1	2	1	1	+1	9	high
Daisuke Ekuni, et al[43]	1	1	1	-	1	1	1	+1	7	high
Akiko Nanri, et al[24]	1	1	1	1	2	1	1	+1	9	high
Tajima Miki, et al[22]	1	1	1	1	1	1	1	+1	8	high
Eiichi Ashizawa, et al[42]	1	1	1	-	1	1	1	+1	7	high
Case-control study	case definition	Representativeness	control selection	Control definition	comparability	assessment of exposure	Same method of ascertainment for cases and controls	Non-Response rate	total	quality evaluation
Radzevičienė, Lina, et al[60]	1	1	-	1	1	1	1	-	6	moderate

Table S4. Assessment of the quality of 1 randomised controlled trial by Cochrane scale.

Study	Random Sequence Generation	Allocation Concealment	Blind	Incomplete Outcome Data	Selective Reporting	Other Sources of Bias
Ford AL, et al[56]	randomisation lists	Unclear	Single blind	Complete	Low risk	Unclear

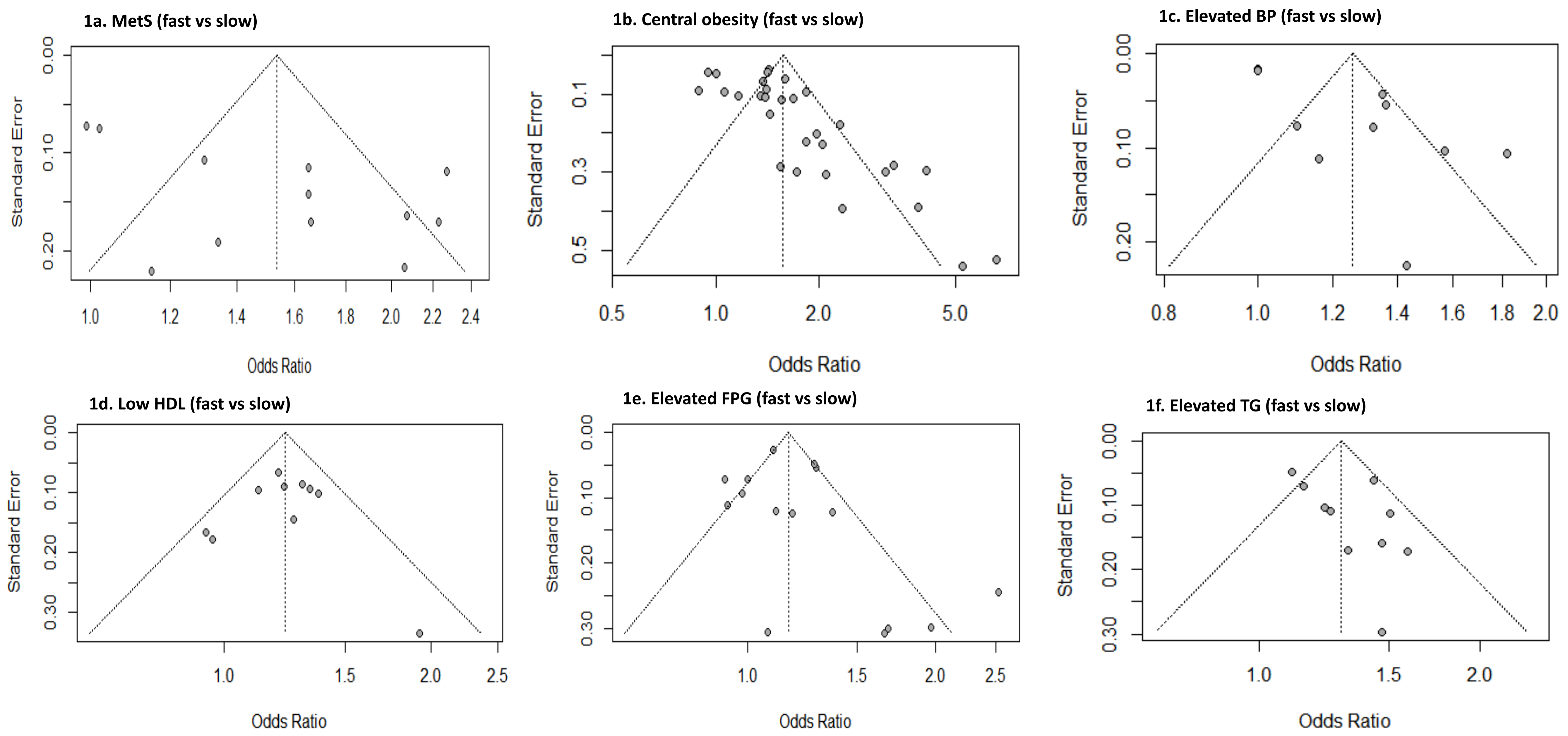

Figure S1. Begg's funnel plots of the included studies in the meta-analysis. (Only when the number of included studies was more than 5, the Begg's test and plot were conducted. MetS, metabolic syndrome; BP, blood pressure; FPG, fasting plasma glucose; TG, triglycerides; HDL, high-density lipoprotein)

Table S5. Begg's test and Egger's test of the included studies in the meta-analysis

Outcomes	Sample size	Eating speed	OR	95%CI	Begg's test P value	Egger's test P value
		slow	Ref			
MetS 7 studies[23,35-40]	32558	faster	1.54	1.27-1.86	0.337	0.014
Central obesity 11 studies[23,24,37,39,44-50]	150868	faster	1.54	1.37-1.73	0.019	0.001
Elevated BP 6 studies[23,24,37,39,50,55]	86783	faster	1.26	1.13-1.40	0.421	0.006
Low HDL 6 studies[23,24,37,39,50,55]	86783	faster	1.23	1.15-1.37	0.929	0.898
Elevated FPG 9 studies[23,24,37,39,50,55,58-60]	286892	faster	1.16	1.06-1.27	0.347	0.056
Elevated TG 6 studies[23,24,37,39,50,55]	86783	faster	1.29	1.18-1.42	0.180	0.115

MetS, metabolic syndrome; BP, blood pressure; FPG, fasting plasma glucose; TG, triglycerides; HDL, high-density lipoprotein.

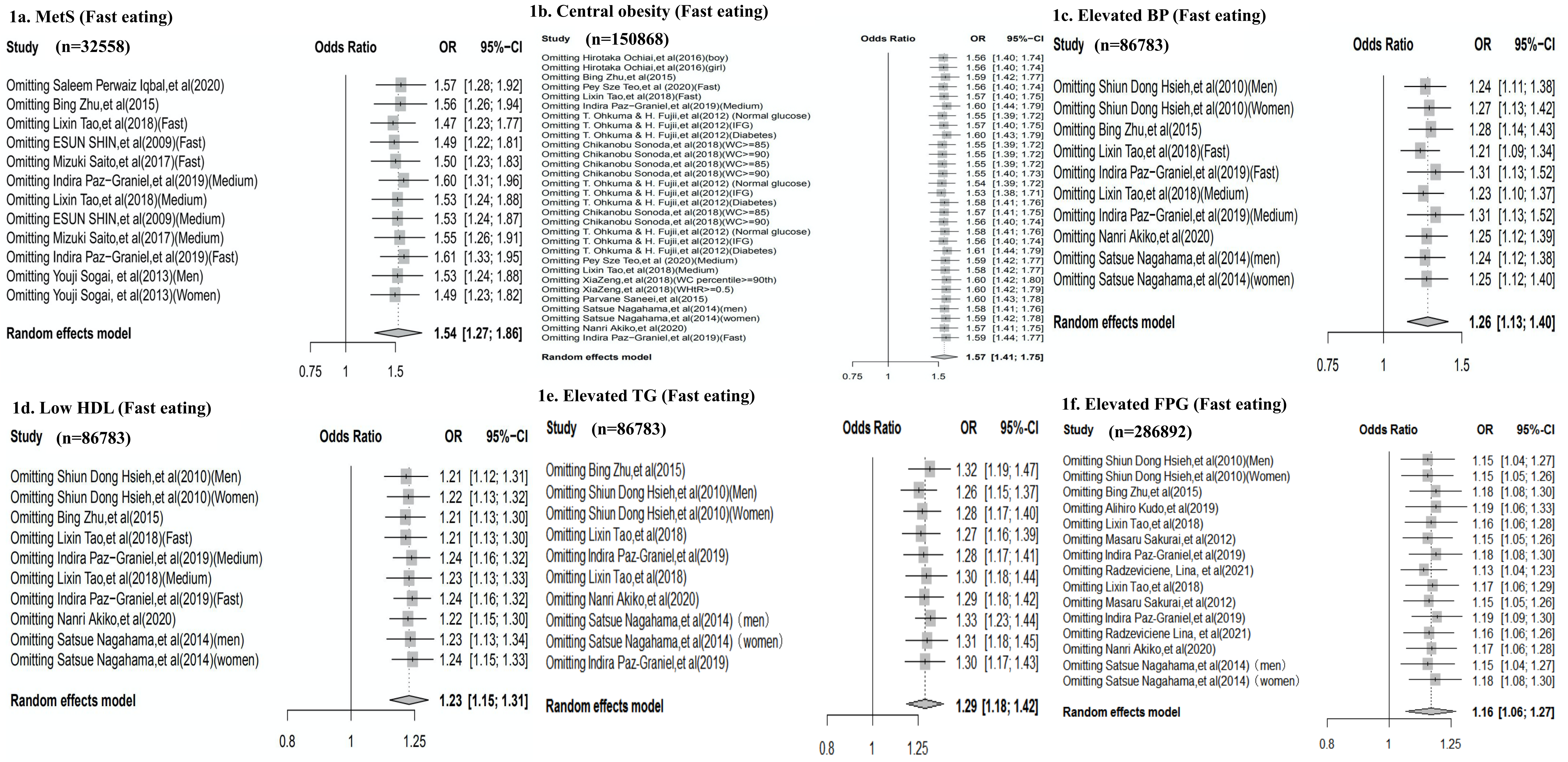

Figure S2. Sensitivity analysis of the included studies in the meta-analysis. (The slow eating group was used as the reference group. MetS, metabolic syndrome; BP, blood pressure; FPG, fasting plasma glucose; TG, triglycerides; HDL, high-density lipoprotein)

Table S6. Meta-regression models of multivariable adjusted analyses

Outcomes	Variables	*P*	Regression coefficients (95% CI)	Tau^2	I^2 residual (%)
MetS	Location	0.003	-0.53(-0.84; -0.22)	0.02	54.32
	Speed	0.371	0.18(-0.24; 0.61)	0.08	86.11
	Study design	0.586	-0.18(-0.90; 0.54)	0.08	87.23
Central Obesity	Location	0.606	-0.04(-0.20; 0.12)	0.13	87.99
	Speed	0.002	0.33(0.13; 0.53)	0.09	87.67
	Study design	0.608	-0.03(-0.54; 0.47)	0.12	88.04
	Gender	0.002	0.72(0.28; 1.17)	0.08	86.31
	Age	0.810	0.17(-1.81; 0.14)	0.12	87.39
Elevated BP	Location	0.010	-0.30(-0.51; -0.01)	0.01	58.38
	Speed	0.784	-0.04(-0.35; 0.27)	0.04	93.53
	Study design	0.729	-0.07 (-0.49; 0.36)	0.04	93.52
Elevated TG	Location	0.566	0.07(-0.22; 0.37)	0.01	54.66
	Speed	0.139	0.21(0.06; 0.36)	0.01	35.24
	Study design	0.426	-0.20(-0.400; 0.01)	0.01	53.95
Elevated FPG	Location	0.942	-0.12(-0.33; -0.31)	0.03	70.61
	Speed	0.719	0.05(-0.23; 0.33)	0.03	74.09
	Study design	0.239	-0.13(-0.10; 0.35)	0.03	74.03
	Gender	0.003	0.35(0.14; 0.56)	0.01	62.66

MetS, Metabolic syndrome; BP, blood pressure; FPG, fasting plasma glucose; TG, triglyceride.
